# CORO1C, a novel PAK4 binding protein, recruitsphospho-PAK4 at serine 99 to the leading edge and promotes the migration of gastric cancer cells: CORO1C recruits PAK4 to the membrane of gastric cancer cells

**DOI:** 10.3724/abbs.2022044

**Published:** 2022-05-18

**Authors:** Xiaodong Li, Min Chen, Ying Yuan, Jiabin Li, Feng Li

**Affiliations:** Department of Cell Biology Key Laboratory of Cell Biology National Health Commission of the PRC and Key Laboratory of Medical Cell Biology Ministry of Education of the PRC China Medical University Shenyang 110122 China

**Keywords:** PAK4, CORO1C, RCC2, microtubule, migration, gastric cancer cell

## Abstract

Gastric cancer is one of the malignant tumors in the world. PAK4 plays an important role in the occurrence and development of gastric cancer, especially in the process of invasion and metastasis. Here we discover that CORO1C, a member of coronin family that regulates microfilament and lamellipodia formation, recruits cytoplasmic PAK4 to the leading edge of gastric cancer cells by C-terminal extension (CE) domain of CORO1C (353–457 aa). The localization of PAK4 on the leading edge of the cell depends on two necessary conditions: the phosphorylation of PAK4 on serine 99 and the binding to the CE domain of CORO1C. Unphosphorylated PAK4 on serine 99 is closely associated with microtubules by PAK4/GEF-H1/Tctex-1 complex. Once phosphorylated, PAK4 is released from microtubule, and then is recruited by CORO1C to the leading edge and regulates the CORO1C/RCC2 (regulator of chromosome condensation 2) complex, leading to the migration of gastric cancer cells. Our results reveal a new mechanism by which PAK4 regulates the migration potential of gastric cancer cells through microtubule-microfilament cross talk.

## Introduction

Gastric cancer is a deadly disease with poor overall survival statistics throughout the world. The majority of new diagnoses per year of gastric cancer occur mainly in Asian and South American countries [1]. One of the major reasons for its relatively poor prognosis is invasion and metastasis, which depends on the dynamic regulation of cytoskeleton. P21-activated kinase 4 (PAK4), a representative member of class II in the PAK family of serine/threonine kinases, is originally identified as a cytoskeleton regulatory protein [2] and plays key roles in cell survival, anchor-independent growth, and the formation of filopodia [ 2– 7], especially in tumorigenesis and development of gastric cancer [ 8– 10] and other malignant tumors [ 6, 11– 18]. As a kinase, PAK4 functions by phosphorylating its downstream substrates including GEF-H1, a microtubule-associated guanine nucleotide exchange factor (GEF). PAK4-GEF-H1 complex associates with microtubule, and phosphorylation of microtubule-bound GEF-H1 by PAK4 releases it into the cytoplasm [19]. Tumor cell migration is a complex biological process in which microfilament together with its binding proteins such as cofilin and Arp2/3 plays an important role in the formation of lamellipodia, which determines the direction of cell movement [ 20, 21]. PAK4 is involved in this process. There is evidence that PAK4 can bind to and phosphorylate LIMK1 to cause cofilin to be phosphorylated and inactivated, losing its function of cutting microfilaments, and stabilizing microfilaments in cells to participate in cell morphology maintenance and cell movement [22]. The vast majority of the data indicate that PAK4 is primarily localized in the cytoplasm. As more data on PAK4 and its partners become available, there are also data revealing the plasma membrane localization of PAK4. It has been reported that PAK4 binds and phosphorylates Integrin β _v_ to promote cell migration [23]. In addition, PKD1 phosphorylates serine 99 of PAK4 and participates in cell migration by forming a PKD1/PAK4/LIMK1 complex [24].

CORO1C, a type I member of the coronin family [25], is widely expressed in a variety of tissues and organs [ 26– 29]. CORO1C locates in areas where the dynamic changes of cell microfilaments are active, such as lamellipodia or membrane ruffles [30], and is involved in dynamic regulation of actin networks and further microfilament-related cell motility [31]. CORO1C is differentially expressed in various solid tumors, such as glioblastoma cancer [32], hepatocellular cancer [33], breast cancer [34], lung cancer [35], colorectal cancer [36] and gastric cancer [37]. Coronins comprise a conserved β-propeller that includes an actin-binding site and acts as a protein scaffold, a linker region that is unique to each coronin which is also known as U region that can be used as the second actin binding region [38] and can bind to RCC2 (regulator of chromosome condensation 2) [39], and a coiled-coil domain that binds Arp2/3 [ 25, 30, 38, 40].

In this study, we explored the molecular mechanism by which Ser99-phosphorylated PAK4 is released from GEF-H1/Tctex-1 complex on microtubule and is recruited to the leading edge of the gastric cancer cell by binding to CE domain of CORO1C. Both PAK4 phosphorylation on serine 99 and recruitment by CORO1C are necessary for PAK4 localization to the leading edge of the cell.

## Materials and Methods

### Cell culture and cell transfection

The human gastric cancer SGC-7901 and BGC-823 cell lines (Cell Bank of Type Culture Collection of Chinese Academy of Sciences, Shanghai, China) were cultured in Dulbecco’s modified Eagle’s medium (DMEM; Sigma-Aldrich, St Louis, USA) containing 10% fetal bovine serum (FBS; Gibco, Carlsbad, USA), 100 U/mL streptomycin and 100 μg/mL penicillin (Invitrogen, Carlsbad, USA). The cells were incubated at 37°C with 5% CO _2_ in a humidified incubator. Lipofectamine 3000 (Invitrogen) was used for transfection according to the manufacturer’s instructions. Forty-eight hour after transfection, the cells were collected for subsequent experiments. PAK4-lentivirus and PAK4-RNAi-lentivirus were purchased from Shanghai GeneChem Company (Shanghai, China). The shRNA PAK4 sequence was 5′-CTTCATCAAGATTGGCGAG-3′ [41]. The shRNA GEF-H1 sequence was 5′-AACAAGAGCATCACAGCCAAG-3′, the siRNA RCC2 sequence was 5′-GACUGAGAAAGAGAAGAUCAATT-3′ (Shanghai GeneChem Company). Commercial lentivirus was used to infect SGC-7901 cells in a 12-well plate with 3 mg/mL polybrene. Infected SGC-7901 cells were identified by western blot analysis.

### DNA constructs

GFP-, DsRed-, Flag-, and GST-tagged *PAK4* were constructed by polymerase chain reaction (PCR) and sub-cloned into pEGFP-C1, DsRed, pcDNA3.1-Flag (Invitrogen), and pGEX-5X-1/2 (GE Healthcare, Bethesda, USA) vectors, respectively. The full length CORO1C was amplified by PCR using cDNA derived from SGC-7901 cells as a template and cloned into pEGFP-C1, DsRed, pcDNA3.1-Flag and pGEX-4T-2 vectors. CORO1C deletion constructs were obtained by PCR and cloned into pGEX-4T-2. The site-directed mutagenesis (PAK4S99A or PAK4S99D) was generated from PAK4 WT using the QuikChange kit (Stratagene, La Jolla, USA) according to the manufacturer′s instructions. eYFP-RCC2 was a kind gift from Prof. Sheng Xiao of Harvard Medical School (Boston, USA).

### Immunofluorescence assay

SGC-7901 cells were seeded onto glass slides at an initial density of 2×10 ^4^ cells/cm ^2^. Then cells were washed with PBS for three times before being fixed in 4% paraformaldehyde (PFA) for 20 min at room temperature. Following three times wash with PBS, cells were permeabilized with 0.1% Triton X-100 for 10 min at room temperature and blocked with normal goat serum for 30 min. Cells were incubated overnight with the primary antibody at 4°C, and incubated with goat anti rabbit/mouse Alexa Fluor 488 (1:500; Invitrogen) and/or Alexa Fluor 546 (1:500; Invitrogen) antibodies for 2 h at room temperature. F-actin, microtubule and nuclei were stained together with secondary antibodies by incubating with phalloidin (FITC labeled, 1:10,000; coumarin labeled, 1:10,000, from Sigma-Aldrich), Paclitaxel Oregon Green 488 (1:1000; Thermo Fisher Scientific, Waltham, USA) and DAPI (1:200,000; Sigma-Aldrich), respectively, in blocking solution. The confocal scanning analysis was performed with an Ultraview Vox Spinning disc confocal microscope (Perkin Elmer, Boston, USA) or FV100 microscope (Olympus, Tokyo, Japan). Fluorescence intensity on the drawn line was analyzed by ImageJ software.

Dithiobis succinimidylpropionate (DSP; 1 mM in PBS, at 37°C, 20 min) was used in the *in vivo* cross-linking experiments, followed by quenching in microtubule-stabilizing buffer (MTSB; 1 mM EGTA, 4% PEG8000, 100 mM PIPES, and 50 mM Glycine, pH6.9) for 15 min at 37°C. Then the cells were incubated with 0.5% DOTMAC (dodecyltrimethylammonium chloride)+1% PFA at 4°C for 5 min, followed by incubation with 1% PFA at 4°C for 20 min [42]. After 3 times wash with PBS, cells were blocked as normal and incubated with α-acetylated tubulin (1:200; Sigma-Aldrich) as primary antibody.

### Western blot analysis

Cells were lysed in lysis buffer containing 0.1 M Tris-HCl, pH 7.0, 2% SDS, 10% glycerol, 0.1 mM DTT, 1 mM EDTA, 1 mM EGTA, 2.5 mM sodium pyrophosphate, 1 mM β-glycerolphosphate, 1 mM sodium orthovanadate, 2 μg/mL leupeptin, and 1 mM PMSF, followed by sonication. The cell lysates were collected and cleaned by centrifugation at 13,000 *g* for 15 min at 4°C. Proteins were resolved by SDS-PAGE and transferred to PVDF membranes (Millipore, Burlington, USA). After being blocked with 5% non-fat milk in TBST for 1 h at room temperature, membranes were incubated with the following primary antibodies in TBST (150 mM NaCl, 5 mM Tris, and 1% Tween-20): anti-CORO1C (1:1000; Abcam, Cambridge, UK), GEF-H1 (1:1000; Cell Signaling, Beverly, USA), PAK4 (1:2000; Cell Signaling), RCC2 (1:1000; Abcam), Flag-tag M2 (1:5000; Sigma-Aldrich), GFP-tag (1:5000; GenScript, Nanjing, China), and GAPDH (1:2000; KangChen, Shanghai, China) antibodies overnight at 4°C. Then the PVDF membranes were incubated with HRP-conjugated goat anti-mouse IgG (1:10,000; Thermo Fisher Scientific) or goat anti-rabbit IgG (1:10,000; Thermo Fisher Scientific) for 1 h at room temperature. After incubation with ECL Plus Western Blotting Substrate (Thermo Fisher Scientific), the immunoblots were developed by Chemiluminescent Imaging System (Tanon, Shanghai, China).

Membrane proteins were extracted using the Minute ^TM^ Plasma Membrane Protein Isolation Kit (Inventbiotech, Plymouth, USA) according to the manufacturer’s recommendation. The extracts were collected and their protein concentrations were determined. Samples containing 10 μg protein were analyzed by western blot analysis. Anti-Na ^+^/K ^+^-ATPase antibody (1:500; Cell Signaling) was used to check for equal loading of membrane proteins.

### Immunoprecipitation assay

The cells were washed twice with cold PBS before lysed in lysis buffer (25 mM Tris, pH 7.6, 150 mM NaCl, 1% Nonidet P-40, and 1 mM EDTA), supplemented with proteinase and phosphatase inhibitors (2 mM dithiothreitol, 1 mM phenylmethylsulfonyl fluoride, 10 μg/mL leupeptin, 10 μg/mL aprotinin, 20 mM glycrophosphate, and 1 mM Na _3_VO _4_). The cell lysates were collected and cleaned by centrifugation at 13,000 *g* for 15 min at 4°C. For immunoprecipitation, 30 μL of 50% protein A agarose slurry (GE Healthcare, Uppsala, Sweden) preloaded with antibodies or normal IgG were added to the equal amount of lysates and rotated overnight at 4°C. The immune complexes were precipitated by centrifugation at 500 *g* for 5 min at 4°C, and washed three times with lysis buffer. The precipitated proteins were mixed with equal volume of 2× SDS-PAGE loading buffer and resolved by SDS-polyacrylamide gel electrophoresis. Cell extracts containing 20 μg total protein were used as inputs. Western blot analysis was finally performed using corresponding antibodies.

### GST pull-down assay

pcDNA3.1 constructs with Flag tag were translated *in vitro* by using the TNT-coupled transcription and translation system (Promega, Madison, USA) following the manufacturer’s instruction. GST constructs were transformed into *Escherichia coli* BL21 cells and the protein expression was induced by IPTG (up to 1 mM) at 37°C for 4 h. The GST or GST fusion proteins were extracted and immobilized onto Glutathione Sepharose 4B beads (Amersham Biosciences, Piscataway, USA) at 4°C for 4 h. Equal amounts of GST and GST-fusion proteins were incubated with *in vitro* translated protein at 4°C overnight. After centrifugation and three times wash with lysis buffer, the beads were eluted with 30 μL of 1×SDS loading buffer and then boiled for 10 min, followed by western blot analysis using anti-Flag-tag M2 antibody (1:5000) as the primary antibody. Ponceau stain indicated the loading of GST or GST-fusion proteins.

### Cell migration and invasion assay

Cell migration and invasion assays were performed using matrigel-coated (for the invasion assay; BD Biosciences, San Diego, USA) or uncoated (for the migration assay) 24-well transwell plates (Corning, Steuben County, USA; 8 μm pore size). SGC-7901 cells in culture medium containing 1% FBS were seeded in the upper chamber, and the lower chamber were filled with culture medium supplemented with 10% FBS. The cells were incubated for 24 h at 37°C. Then the cells on the upper surface of membrane were removed with a cotton swab, and the migrated/invaded cells were fixed with 95% ethanol for 20 min. The fixed cells were stained with 4% trypan blue for 20 min, after which the membrane was washed with PBS for 3 times and mounted. Images were captured and the number of cells was counted under a microscope. All assays were performed in triplicate.

### Wound healing assay

For the wound healing assay, 2×10 ^5^ cells were seeded into each well of 12-well plates with three replicates per group, and incubated to reach confluence after transfection. The monolayer was scratched using a tip and washed with serum-free DMEM to remove any debris. Then the cells were cultured in complete medium. Cells were photographed at indicated time pionts. The closure area of wound was calculated as follows: migration area (%)=(A _0_−A _n_)/A _0_×100%, where A _0_ represents the area of initial wound area, A _n_ represents the remaining area of wound.

### Statistical analysis

Data were expressed as the mean±standard deviation (SD) and analyzed using IBM *SPSS* software version 26.0 (Chicago, USA). Two-sided Student’s *t*-test was used to determine the significant difference of migration. The *P*-values less than 0.05 were considered statistically significant.

## Results

### CORO1C interacts with PAK4 through its CE domain

To search for novel interacting proteins of PAK4 related to its membrane localization, Flag-tagged PAK4 was overexpressed in SGC-7901 cells, and the lysate was immunoprecipitated using anti-α-Flag antibody. The immunoprecipitates were analyzed by mass spectrometry, and the result showed that CORO1C, a member of the crown protein family, was an interesting hit ( Supplementary Table S1).

**Figure 1:**
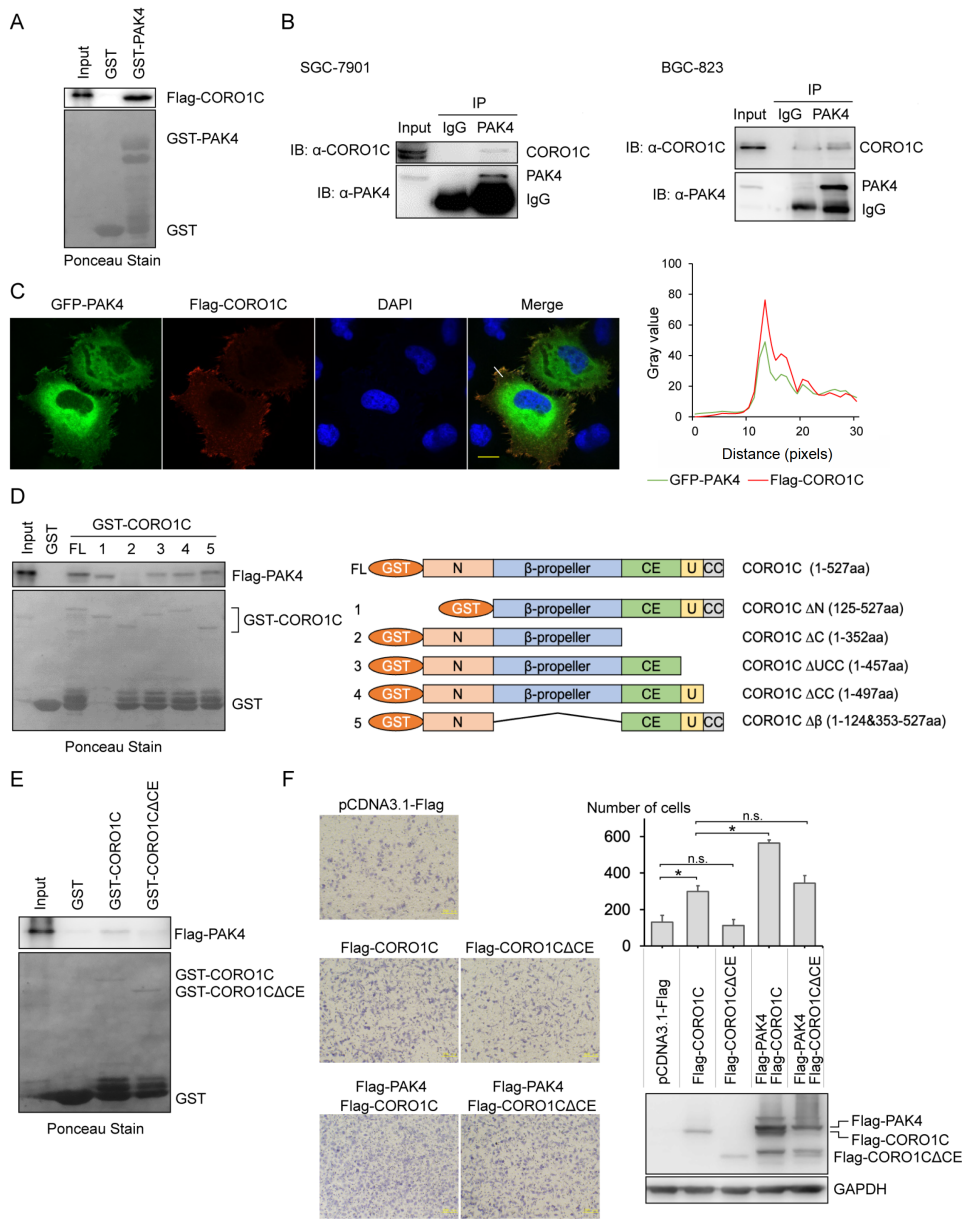

To confirm the interaction between CORO1C and PAK4, GST pull-down assay was performed, and the result showed that *in vitro*-translated CORO1C bound with GST-PAK4 but not with GST along ( Figure 1A). The endogenous immunoprecipitation assay was then performed to explore the intracellular interaction between PAK4 and CORO1C in SGC-7901 cells and BGC-823 cells ( Figure 1B). It was reported that PAK4 localizes to the leading edge of the cell [24]. Immunofluorescence assay showed that GFP-tagged PAK4 co-localizes with CORO1C on the membrane of SGC-7901 cells ( Figure 1C). ImageJ software was used to analyze the fluorescence distribution. Along the line drawn, the gray value changes of the fluorescence are synchronized, indicating the reliability of the co-localization of the two proteins. To map the PAK4-binding region of CORO1C, we constructed a series of GST-fused CORO1C truncations, among which only those with CE domain (353–457 aa) can pull down PAK4 ( Figure 1D). This was confirmed by GST pull-down assay using GST-tagged CORO1C∆CE as the bait ( Figure 1E). Either PAK4 or CORO1C was reported to be involved in cell migration. In order to detect the effect of the interaction between PAK4 and CORO1C on the migration of gastric cancer cells, transwell assay was performed. The result showed that overexpression of CORO1C promoted the migration of gastric cancer cells, but not CORO1CΔCE. The migration capability of the cells that overexpress CORO1C was correspondingly enhanced after further overexpression of PAK4, which was not found in cells that overexpress CORO1CΔCE ( Figure 1F). These findings suggest that the interaction of PAK4 with CORO1C facilitates the migration of gastric cancer cells.

### PAK4 is required for the CORO1C/RCC2 complex

**Figure 2:**
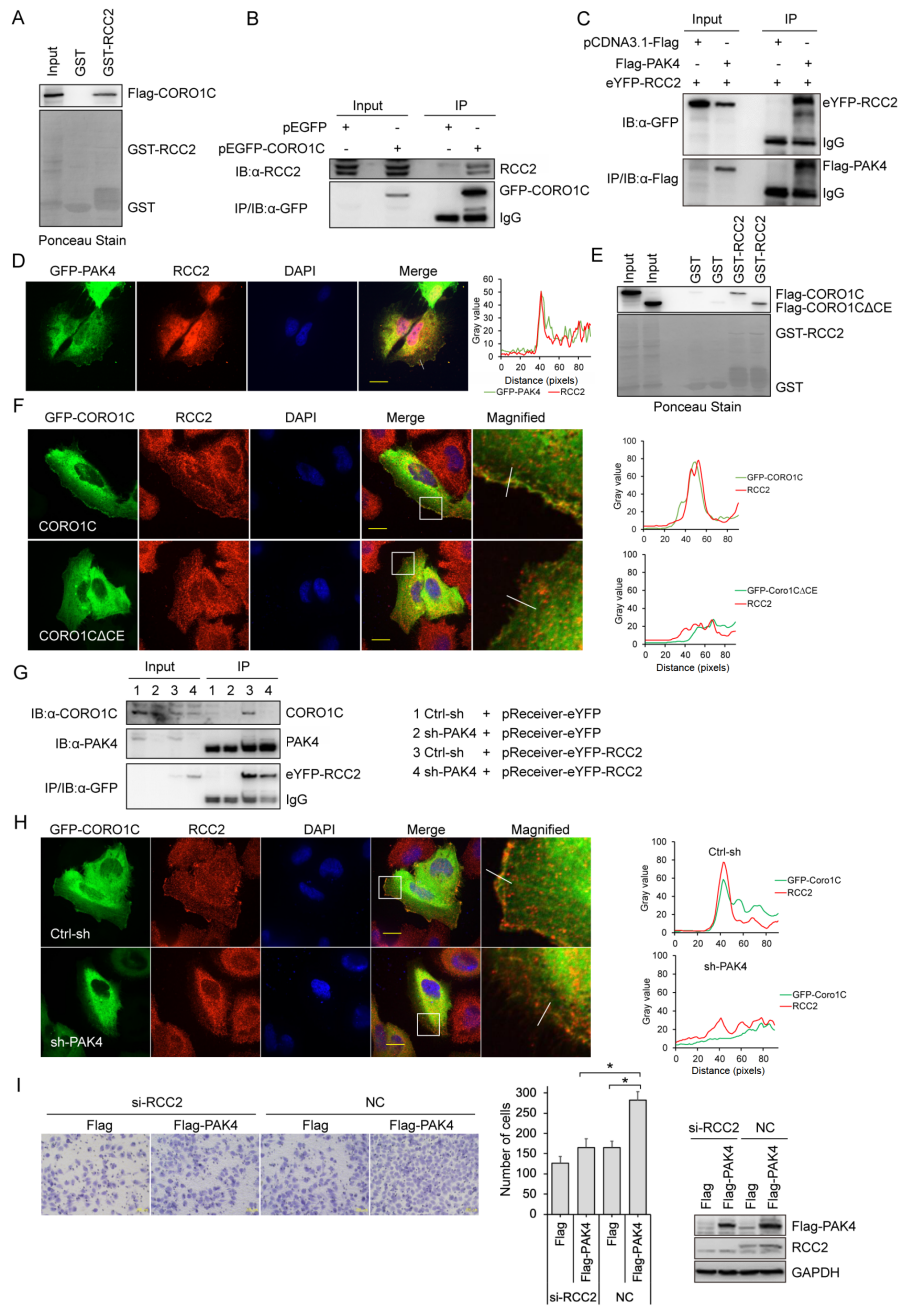

It has been reported that RCC2 binds to the 351–435 aa region of CORO1C [38], which overlaps with the PAK4-binding region that we have demonstrated. This prompted us to reveal the competition between RCC2 and PAK4 in binding with CORO1C. The binding of RCC2 with CORO1C was confirmed by GST pull-down assay ( Figure 2A) and their interaction in SGC-7901 cells was also verified by immunoprecipitation assay ( Figure 2B). It was also found that PAK4 co-immunoprecipitated ( Figure 2C and Supplementary Figure S1A) and colocalized with RCC2 ( Figure 2D) in SGC-7901 gastric cancer cells. Interestingly, both full length and CE deletion of CORO1C were pulled down by GST-RCC2 ( Figure 2E), indicating that additional RCC2-binding site on CORO1C and the relationship between PAK4 and RCC2 might be more complicated than competition. GST pull-down assay showed that though RCC2 interacted with CORO1C∆CE, the membrane co-localization of RCC2 with CORO1C∆CE disappeared ( Figure 2F), while further PAK4 knockdown also ceased both the interaction of RCC2 with CORO1C ( Figure 2G) and the leading edge co-localization of RCC2 in gastric cancer cells ( Figure 2H). All the above data indicate that PAK4 plays key roles in CORO1C and RCC2 complex and distribution. We further determined whether the interaction between PAK4 and RCC2 is necessary for the migration of gastric cancer cells by transwell assay using cells with knockdown of *PAK4* or *RCC2*. The results showed that knockdown of *RCC2* attenuated the increased migration of SGC-7901 cells induced by overexpression of PAK4 ( Figure 2I). Undoubtedly, knockdown of *RCC2* was unable to further attenuate the migration of SGC-7901 cells with stable *PAK4* silencing ( Supplementary Figure S1B). These results indicate that PAK4 is necessary for RCC2 to promote the migration of gastric cancer cells.

### Phosphorylation of PAK4 on serine 99 is necessary for its localization on cell membrane

**Figure 3:**
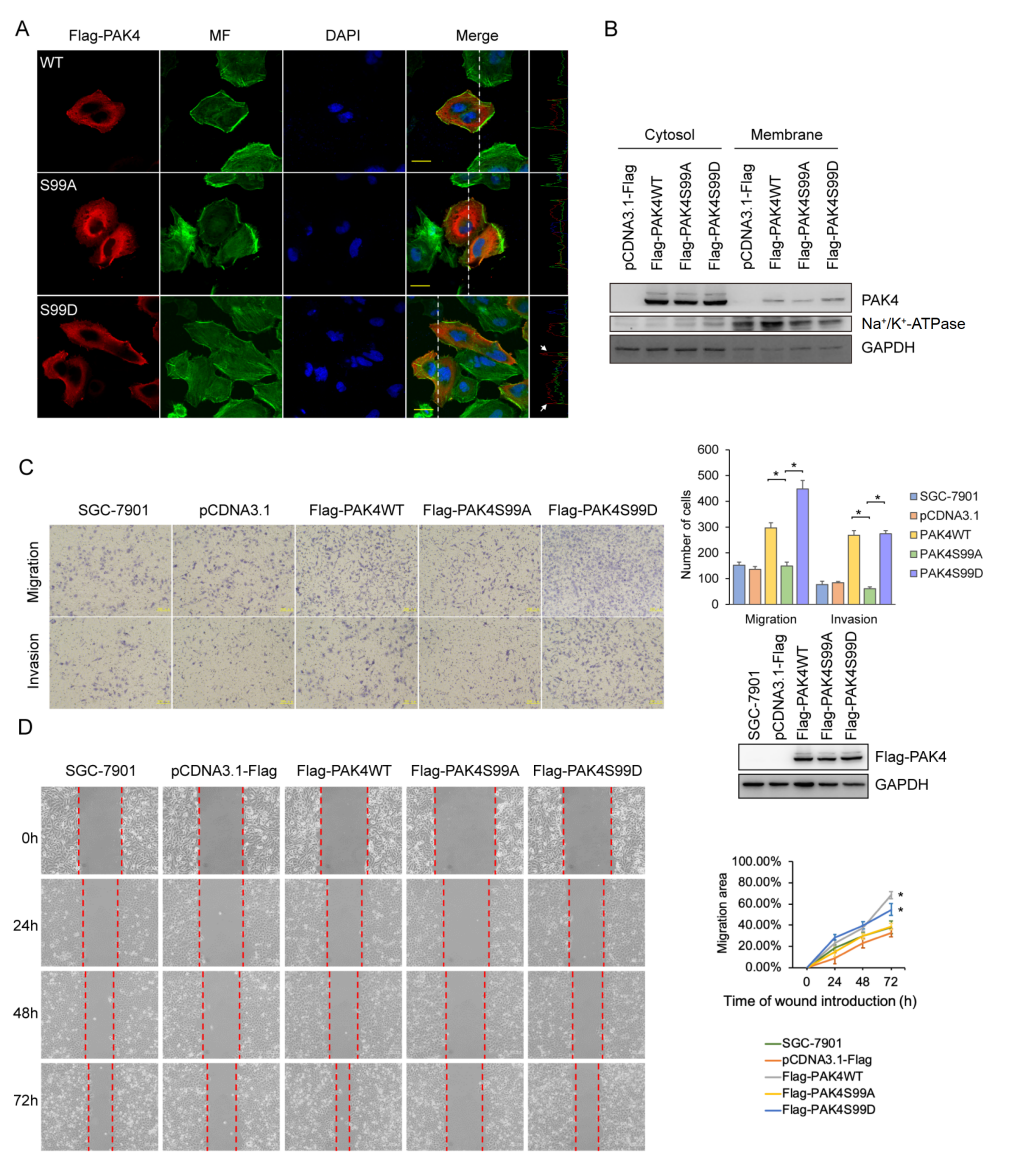

By analyzing the distribution of PAK4 in gastric cancer cells, we found that a small amount of PAK4 co-localized with CORO1C on the plasma membrane, while others located around the nucleus away from the plasma membrane, suggesting a complicated regulatory mechanism for the distribution of PAK4 in gastric cancer cells. PKD1 phosphorylates PAK4 on both Ser99 and Ser474, among which Ser99 is necessary for the localization of PAK4 to the leading edge [24]. To evaluate the contribution of Ser99 residue to the distribution of PAK4 in cells and to the migration of gastric cancer cells, we constructed two PAK4 mutants: PAK4S99A (Ser to Ala) that cannot be phosphorylated and PAK4S99D (Ser to Asp) that mimic the phosphorylation state. As expected, PAK4S99D was localized to the plasma membrane of gastric cancer cells, but not PAK4S99A ( Figure 3A). To further confirm this, cell membrane fractions from gastric cancer cells overexpressing PAK4 wild-type or Ser99 mutants were prepared. Western blot analysis results revealed that the level of PAK4S99D in plasma membrane fraction of gastric cancer cell was much higher than that of PAK4S99A ( Figure 3B). PAK4S99A-overexpressing gastric cancer cell showed weaker migration and invasion capabilities than cells overexpressing PAK4S99D or wild-type PAK4, as revealed by transwell and wound healing assays ( Figure 3C,D). All these data suggest that the phosphorylation state of the Ser99 residue of PAK4 regulates the distribution of PAK4 in gastric cancer cells as well as the migration of gastric cancer cells.

### CORO1C recruits phospho-PAK4 (Ser99) to leading edge of cells via CE domain of CORO1C

**Figure 4:**
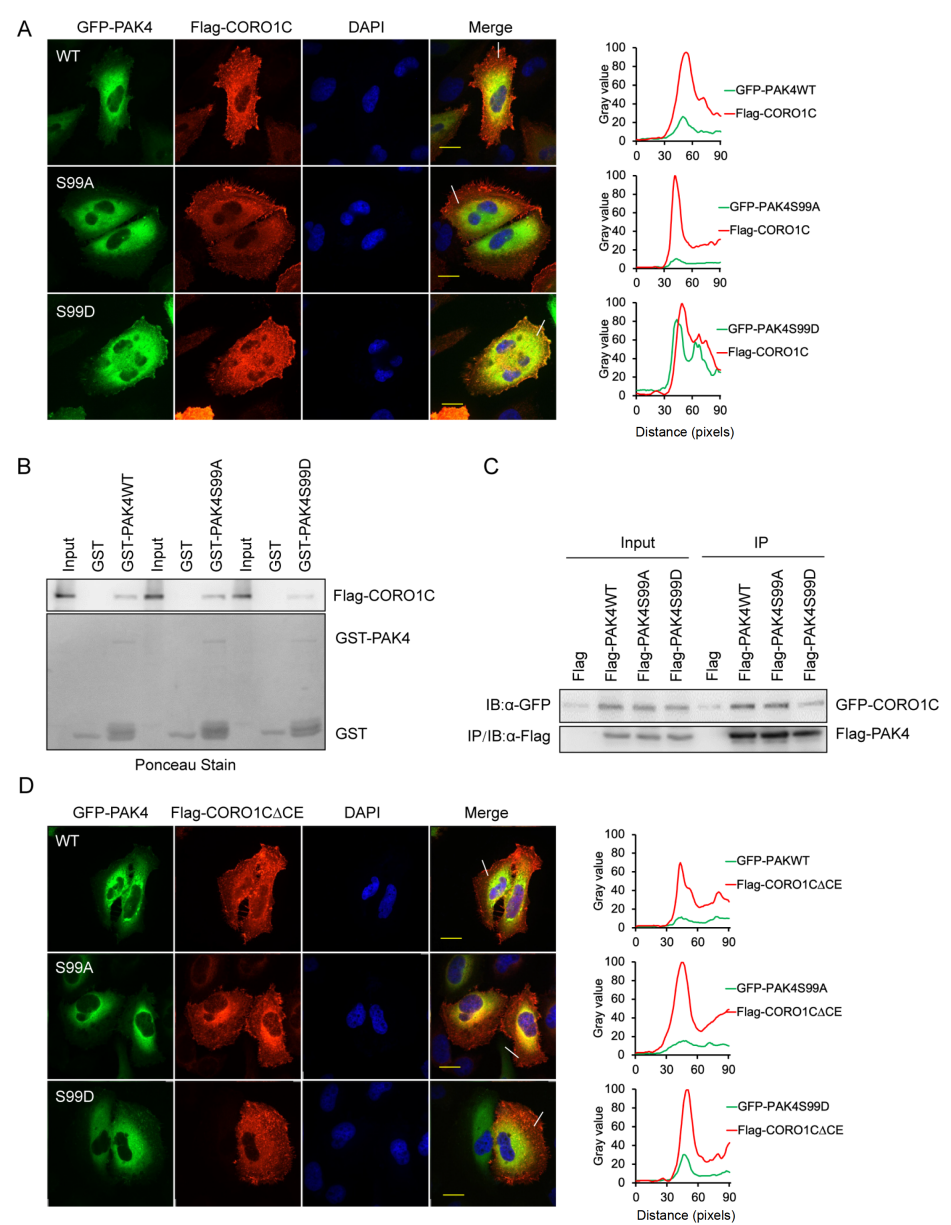

To further evaluate the role of Ser99 phosphorylation of PAK4 in its membrane localization, the co-localization of PAK4 mutants with CORO1C was examined. As expected, PAK4S99D showed significant membrane co-localization with CORO1C, but not PAK4S99A ( Figure 4A). Therefore the relationship between the phosphorylation status of Ser99 and the interaction of CORO1C with PAK4 were further explored. However, both GST pull-down and Co-IP results revealed the interaction of CORO1C with both PAK4S99D and PAK4S99A ( Figure 4B,C). Furthermore, when CORO1C∆CE was overexpressed in gastric cancer cells, the localization of PAK4S99D in the plasma membrane was significantly reduced compared with gastric cancer cells overexpressing wild-type CORO1C ( Figure 4A,D).This result suggested that although recruitment of PAK4 to the plasma membrane depends on both Ser99 phosphorylation and CE domain of CORO1C, there must be other mechanisms which prevent non-phosphorylated PAK4 from being recruited by CORO1C.

### PAK4S99A is associated with microtubules in gastric cancer cells.

**Figure 5:**
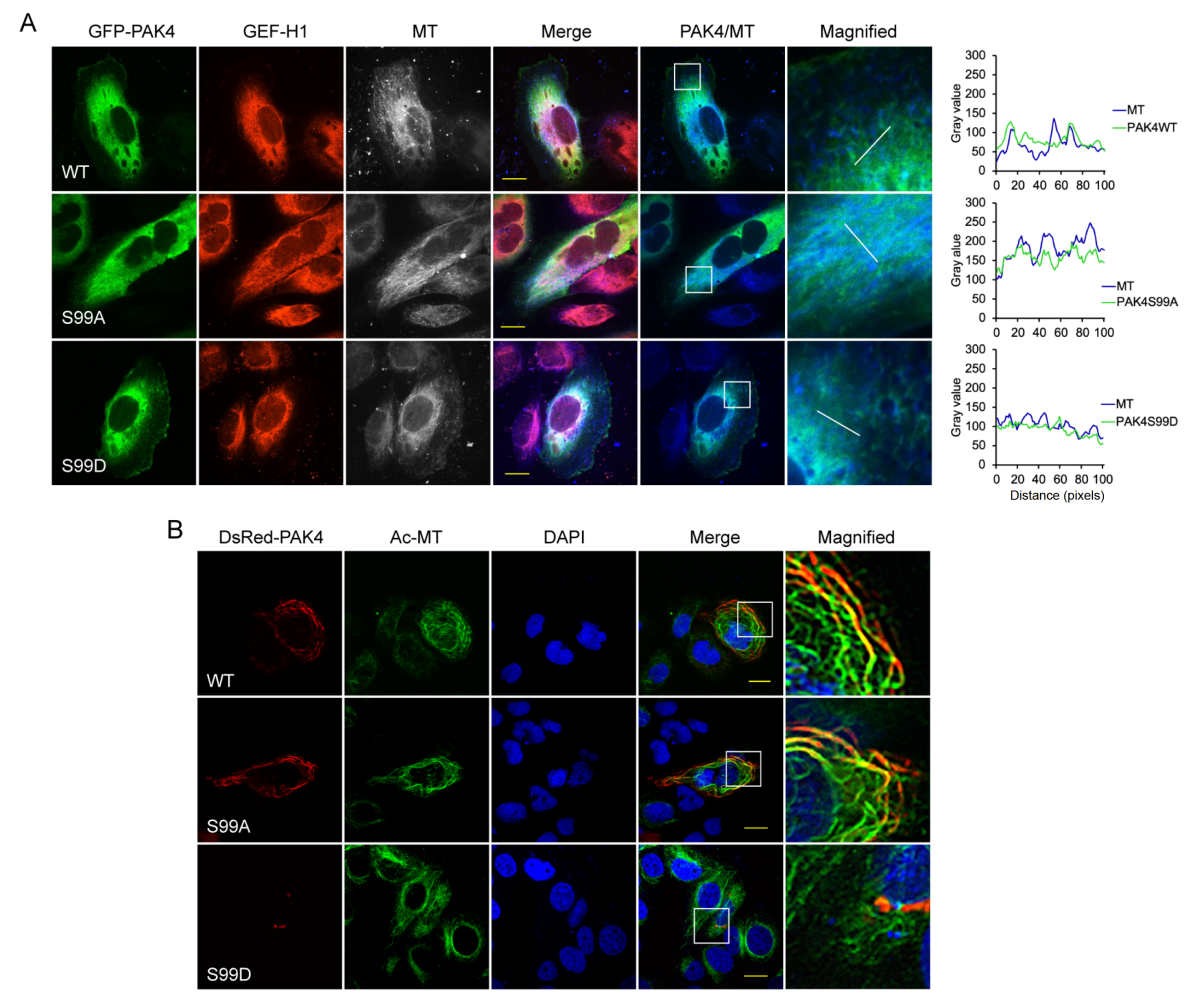

It has been reported that PAK4 forms a stable complex with GEF-H1 and associates with microtubules [19]. We then examined the localization of PAK4 mutants and GEF-H1 in SGC-7901 cells. It was found that PAK4 co-localized with GEF-H1, and PAK4S99A showed obvious association with microtubules compared with PAK4S99D in gastric cancer cells ( Figure 5A). To further confirm this, we used a dithiobis succinimidylpropionate (DSP)-fixative procedure to preserve cytoskeletal structures and pre-form any spatially associated or microtubule-associated proteins in cells prior to conventional fixation [42]. Dodecyltrimethylammonium chloride (DOTMAC) was used instead of Triton X-100 for solubilization. This enabled us to preserve the association of PAK4 with microtubule. DsRed-PAK4 and acetylated tubulin in SGC-7901 cells were detected by confocal microscopy. The results showed that both PAK4WT and PAK4S99A co-localized with microtubules, but not PAK4S99D ( Figure 5B), indicating that the phosphorylation on Ser99 of PAK4 might be the key reason for the dislocation of PAK4 from microtubules.

### Unphosphorylated PAK4 at Ser99 is restricted on microtubule by the GEF-H1/Tctex-1 complex

**Figure 6:**
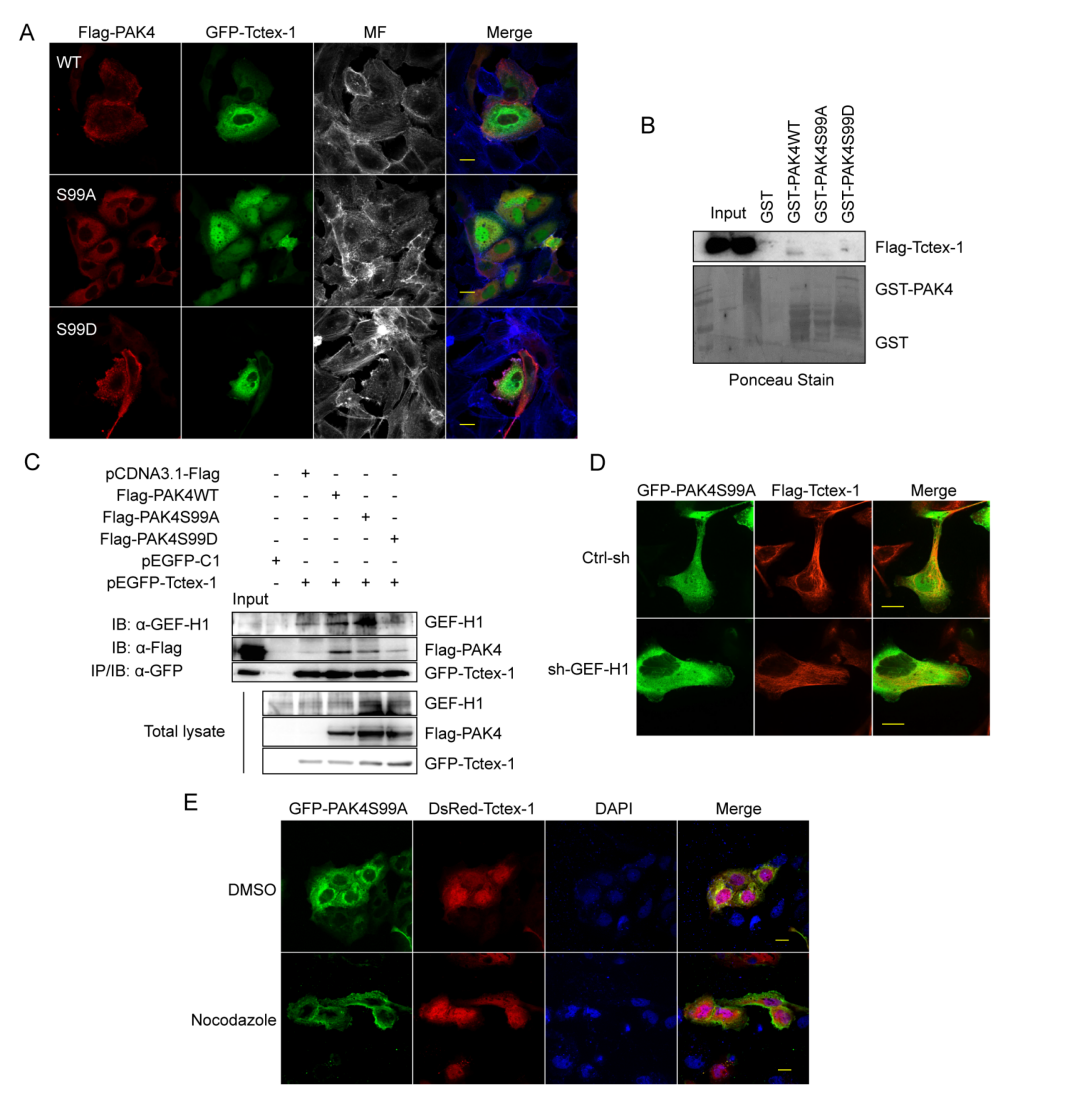

It has been reported that Lfc (murine isoform of GEF-H1) interacts with Tctex-1, the light chain of motor protein dynein [43]. Thus Tctex-1 most probably plays roles in the co-localization of PAK4S99A with microtubules. As expected, the immunofluorescence assay indicated that PAK4S99A co-localized more obviously with Tctex-1 than PAKS99D, with significant co-localization with microfilaments in the pseudopodia of cells ( Figure 6A). Direct binding between bacterially expressed GST-PAK4 and *in vitro* translated Tctex-1 was not found ( Figure 6B), indicating that microtubule association of PAK4 depends on GEF-H1. Thus, co-immunoprecipitationwas carried out to investigate the association of PAK4 with GEF-H1/Tctex-1, and the results showed that both GEF-H1 and PAK4 were co-precipitated with Tctex-1, which was confirmed by western blot analysis. Interestingly, less PAK4S99D and GEF-H1 were co-precipitated with Tctex-1 in PAK4S99D-overexpressing SGC-7901 cells, compared to PAK4S99A ( Figure 6C), indicating that the phosphorylation of PAK4 at Ser99 may release both PAK4 and GEF-H1 from microtubules to the cytoplasm. To confirm this, localization of PAK4S99A and Tctex-1 in *GEF-H1*-knockdown cells was examined, and the results showed that knockdown of *GEF-H1* suppressed the co-localization of PAK4S99A with Tctex-1 ( Figure 6D). Meanwhile, nocodazole treatment decreased the co-localization of PAK4S99A with Tctex-1, and resulted in membrane localization of PAK4S99A ( Figure 6E). Taken together, these results demonstrate that unphosphorylated PAK4 at Ser99 is confined to microtubules by the GEF-H1/Tctex-1 complex, while phosphorylation at Ser99 impairs the affinity of the complex and dissociates PAK4 from microtubules.

## Discussion

Cell motility is essential for many biological processes such as embryonic morphogenesis, immune surveillance, and tissue repair. Dysregulation of cell motility is associated with a number of disease states including metastatic cancer [44]. Tumor cell migration is a complex biological process in which Rac1, cofilin and Arp2/3 play key roles, form lamellipodia, and determine the direction of cell movement [ 20, 21]. As the regulator of membrane protrusion, Rac1 is a key factor in migration signaling and is necessary for cell migration along the extracellular matrix. Lamellipodium formation involves new actin polymerization and requires actin nucleators, such as the Arp2/3 complex, that are activated by Rac [45].

In many cancers, PAK4 is shown to be overexpressed or mutated, and its inhibition by pharmacological means is suggested to target cancer metastasis [ 46, 47]. PAK4 is involved in this process. Evidence suggests that PAK4 binds to and phosphorylates LIMK1, which in turn leads to phosphorylation and inactivation of cofilin. Inactivated cofilin loses its function of severing microfilaments, and participates in cell morphology maintenance and cell movement by stabilizing intracellular microfilaments [22]. To further investigate the mechanism by which PAK4 is involved in the formation of lamellipodia, we performed mass spectrometry to find new targets of PAK4. CORO1C, a member of coronin family, associates with poor survival rates in gastric cancer and promotes metastasis [37]. Interactions among CORO1C, RCC2 and Rac1 focus active Rac1 to a single protrusion, play crucial roles in migration guidance [38].

We confirmed the interaction of CORO1C with PAK4 and found the colocalization of CORO1C with PAK4 on the membrane of gastric cancer cells. The CE domain was mapped to be a necessary region for PAK4 binding. It was also identified to be a necessary region both for the leaing edge localization of PAK4 and for PAK4/CORO1C-induced migration of gastric cancer cells.

CORO1C regulates Rac1 activity via the CORO1C/RCC2/Rac1 complex in which CORO1C redistributes Rac1 from lateral to protrusive membrane and RCC2 limits GEF activation of Rac1 by obscuring the switch regions and thereby prevents the formation of multiple protrusions [38]. Here, we confirmed the interaction between CORO1C and RCC2. It has been reported that RCC2 binds to the linker region (351–435 aa) of CORO1C by using the C-terminal tail (including linker region and coiled-coil domain) as a bait [38], which is almost the same as the CE domain. Unfortunately, CORO1C without the CE domain can still bind with RCC2, indicating an additional binding sequence in the N-terminal region of CORO1C. RCC2, together with PAK4, were co-immunoprecipitated with CORO1C, whereas knockdown of PAK4 ceased the association of CORO1C with RCC2 in gastric cancer cells. Further CE domain deletion of CORO1C decreased the colocalization of CORO1C with RCC2 on the leading edge. All these data indicate that PAK4 is recruited by CORO1C via its CE domain and then regulates the stability of CORO1C/RCC2 complex.

It has been reported that phosphorylation at serine 99 of PAK4 is required for its targeting to the leading edge of the cell [24]. Our results confirmed that phosphorylation at serine 99 distributed PAK4 to the cell membrane, and interestingly, this phosphorylation is also required for CORO1C-promoted migration of gastric cancer cells. Thus, it is necessary to explore whether the recruitment of PAK4 by CORO1C to plasma membrane depends on the phosphorylation at serine 99 of PAK4 or not. As expected, the co-localization of PAK4 with CORO1C on plasma membrane depends on its phosphorylation status of serine 99. On the other hand, both the results of GST-pull down and co-immunoprecipitation proved that the phosphorylation status of PAK4 serine 99 does not affect its interaction with CORO1C. Since it has been reported that phosphorylation on serine 99 of PAK4 does not affect its kinase activity [24], there must be some factors that prevent unphosphorylated PAK4 from localizing to the plasma membrane.

Many studies have implicated the roles of PAKs in the control of the microtubule network [48]. Activated PAK1 has been found to be associated with MTOCs and play a role in microtubule dynamics in neural precursors undergoing mitosis, while overexpression of PAK1 causes mitotic spindle defects in MCF-7 cells [49]. Comparing with PAK1, only few substrates of PAK4 associate with microtubule [ 19, 41], among which GEF-H1 is related most closely to the microtubule. Co-expression of activated PAK4 with GEF-H1 leads to the separation of GEF-H1 from microtubule [19]. These findings lead us to study the relationship between Ser99 modification and microtubule localization of PAK4 in gastric cancer cells. Immunofluorescence microscopy results indicated that PAK4S99A co-localizes with GEF-H1 on microtubule in gastric cancer cells, but not cytoplasmic PAK4S99D. To further confirm this, DSP was used in the immunofluorescence experiments [ 19, 42]. A large amount of PAK4S99A related to the cytoskeleton was retained and showed obvious co-localization with microtubules, compared with PAK4S99D. It has been reported that Lfc, the conserved murine isoform of GEF-H1, is recruited to the microtubule array by binding to Tctex-1 directly [50]. This promoted us to study the association of PAK4 with GEF-H1 and Tctex-1 in gastric cancer cells. PAK4S99A, but not PAK4S99D, co-localized with Tctex-1 in the cytosol of gastric cancer cells. No positive Tctex-1 band was obtained in GST pull-down assay by using GST-PAK4WT, GST-PAK4S99A, or GST-PAK4S99D as the bait. Silencing of *GEF-H1* ceased the colocalization of PAK4S99A with Tctex-1. More GEF-H1 was co-immunoprecipitated with Tctex-1 in PAK4S99A-overexpressing gastric cancer cells than in PAK4S99D-overexpressing gastric cancer cells. Furthermore, depolymerization of microtubule by nocodazole also decreased the co-localization of PAK4SS99A with Tctex-1. Taken together, we demonstrated that unphosphorylated PAK4 on Ser99 is restricted on microtubules by the PAK4/GEF-H1/Tctex-1 complex.

**Figure 7:**
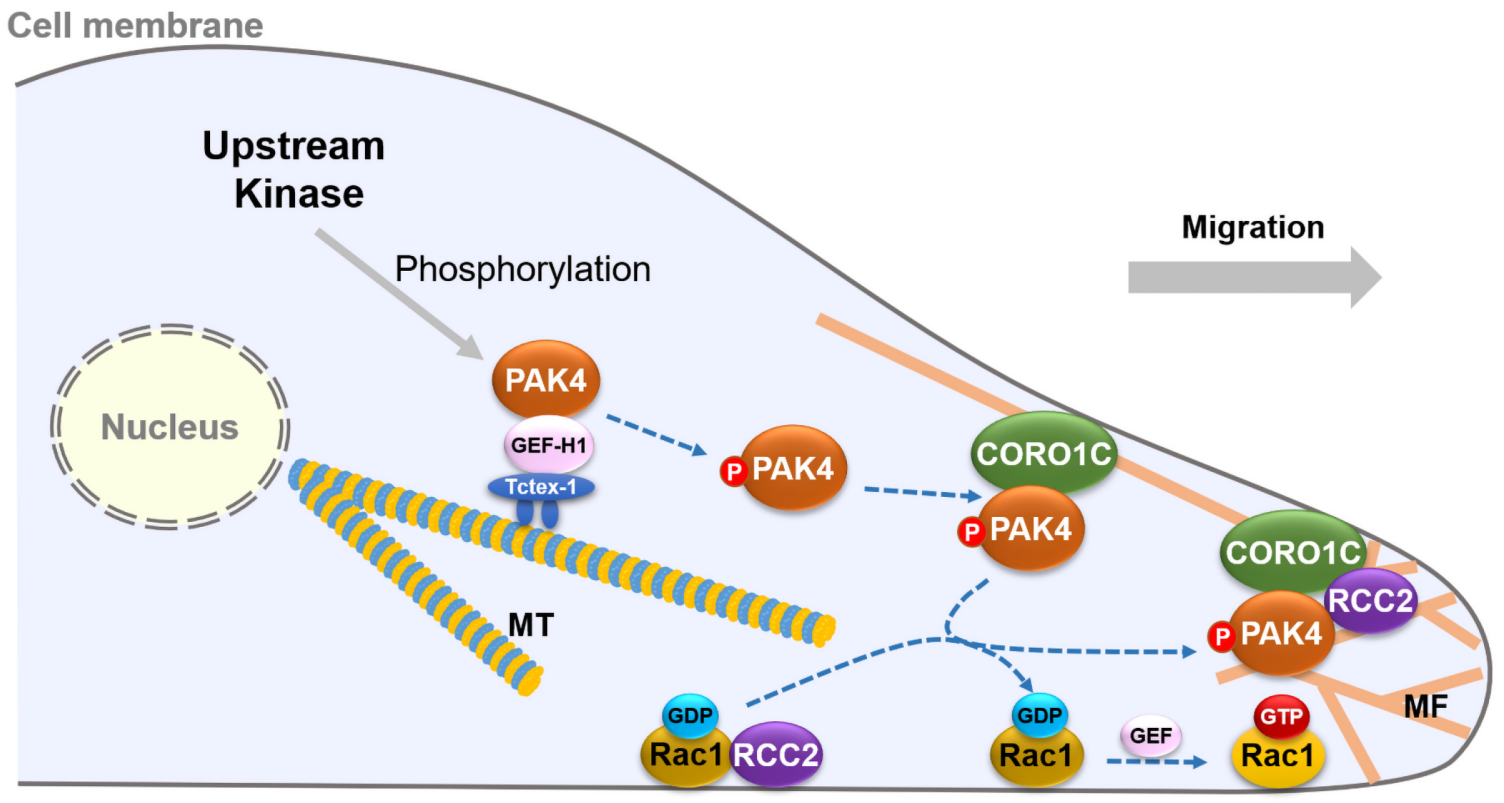

In summary, we report that the potential mechanism of PAK4 recruitment by CORO1C to the plasma membrane depends on the serine 99 phosphorylation. Notably, Unphosphorylated PAK4 on serine 99 is closely associated with microtubules by the PAK4/GEF-H1/Tctex-1 complex. Once phosphorylated, PAK4 is released from microtubules, and then is recruited by CORO1C to the leading edge and regulates the CORO1C/RCC2 complex, leading to the migration of gastric cancer cells. Our results reveal a new mechanism by which PAK4 regulates the migration potential of gastric cancer cells through microtubule-microfilament cross talk ( Figure 7).

## Supplementary Data

Supplementary data is available at *Acta Biochimica et Biophysica Sinica* online.
